# The preventive effect of sodium-glucose co-transporter-2 inhibitors on life-threatening arrhythmias in patients with chronic kidney disease: a meta-analysis

**DOI:** 10.3389/fcvm.2026.1781810

**Published:** 2026-05-13

**Authors:** Qian Yang, Yongjian Jia, Zhuodong Li, Yanmin Yu, Yi Dang

**Affiliations:** 1 Department of Cardiology, Hebei General Hospital, Shijiazhuang, Hebei, China; 2 Hebei Key Laboratory of Precision Medicine Translational Research on Cardiovascular Disease, Hebei General Hospital, Shijiazhuang, Hebei, China

**Keywords:** atrioventricular block, cardiac arrest, chronic kidney disease, sodium-glucose co-transporter-2 inhibitors, ventricular arrhythmia

## Abstract

**Background:**

The main cause of death for chronic kidney disease (CKD) is cardiac arrest caused by life-threatening arrhythmias, such as ventricular arrhythmia (VA) and atrioventricular block (AVB). Sodium-glucose co-transporter-2 inhibitors (SGLT2i) have been proven to have a preventive effect on atrial arrhythmia. This meta-analysis aimed to evaluate whether SGLT2i could prevent life-threatening arrhythmias in CKD patients.

**Methods:**

We searched PubMed, Embase, Cochrane Library, and Clinical Trials.gov until August 2025. Randomized controlled trials (RCTs) that compared the effect of SGLT2i and placebo on cardiac arrest, VA, and AVB in CKD patients were included. VA included ventricular tachycardia (VT), ventricular flutter (VFL), and ventricular fibrillation (VF). AVB only included second-degree and third-degree AVB. Risk ratio (RR) with 95% confidence interval (CI) was calculated using a random-effects model.

**Results:**

8 RCTs were included. Among them, 7 RCTs reported cardiac arrest, 5 reported VA, and 5 reported AVB. SGLT2i could reduce the risk of cardiac arrest in CKD patients (0.12% vs. 0.23%; RR 0.53, 95% CI 0.29–0.95, *P* = 0.03), while didn't reduce the risk of VA (0.09% vs. 0.12%; RR 0.72, 95% CI 0.34–1.55, *P* = 0.40) and AVB (0.15% vs. 0.19%; RR 0.77, 95% CI 0.42–1.40, *P* = 0.38). Subgroup analyses revealed that SGLT2i did not prevent the incidents of VF/VFL (RR 0.42, 95% CI 0.13–1.34, *P* = 0.14), VT (RR 1.20, 95% CI 0.42–3.43, *P* = 0.74), second-degree (RR 2.03, 95% CI 0.65–6.33, *P* = 0.22) and third-degree (RR 0.51, 95% CI 0.24–1.08, *P* = 0.08) AVB.

**Conclusion:**

SGLT2i could reduce the risk of cardiac arrest in CKD patients, but had no effect on specific types of life-threatening arrhythmias, such as VT, VF/VFL, second-degree and third-degree AVB.

**Systematic Review Registration:**

https://www.crd.york.ac.uk/PROSPERO/view/CRD420261388417

## Introduction

Chronic kidney disease (CKD), with a prevalence of 13.4% globally, is defined as an estimated glomerular filtration rate (eGFR) < 60 mL/min/1.73 m^2^ or the presence of kidney damage that has been present for >3 months (1). CKD is a worldwide public health problem, not only reducing patients' life expectancy, but also increasing medical and financial burdens (1, 2).

The main cause of death for CKD is cardiac arrest and sudden cardiac death (SCD) (1, 2). The majority of them are caused by life-threatening arrhythmias (1, 2), mainly ventricular arrhythmia (VA), including ventricular tachycardia (VT), ventricular flutter (VFL), and ventricular fibrillation (VF). In addition, severe bradyarrhythmia, including sinus bradycardia and atrioventricular block (AVB), may directly cause cardiac arrest or lead to secondary life-threatening VA. Although significant progress has been made in pacing, defibrillation, and catheter ablation, anti-arrhythmic drugs as the basic treatment remain a global research challenge, especially for CKD patients.

SGLT2i, as a new glucose-lowering drug, has been proven to have protective effects on the heart and kidney (3). Recent studies showed that the potential mechanisms of SGLT2i on arrhythmias may include suppressing sympathetic hyperactivity, improving cardiac fibrosis and remodeling, modulating cardiac energy metabolism, and so on (4, 5). Our previous meta-analysis showed that SGLT2i could reduce the risk of atrial fibrillation and flutter in CKD patients (6). No meta-analyses investigated the effect of SGLT2i on life-threatening arrhythmias in CKD patients, such as cardiac arrest, VA, AVB, and so on, which often cause hemodynamic disorders and lead to death.

A recent meta-analysis (7) included 39 trials involving 107,770 patients and showed that SGLT2i had no impact on the risk of VT, VF, and sinus bradycardia. However, it did not perform subgroup analyses based on different populations, and did not investigate the effect of SGLT2i on cardiac arrest and AVB. Another meta-analysis (8) showed SGLT2i could reduce the risk of cardiac arrest in CKD patients, but only 3 trials included in the CKD subgroup.

In our article, cardiac arrest, VT, VFL, VF, second-degree and third-degree AVB were defined as life-threatening arrhythmias. This meta-analysis aimed to evaluate whether SGLT2i could prevent these arrhythmias in CKD patients.

## Methods

### Study search and data sources

This meta-analysis was performed according to the Preferred Reporting Items for Systematic Reviews and Meta-Analyses (PRISMA) guidelines (9). PubMed, Embase, Cochrane Library, and Clinical Trials.gov were searched until August 2025. The search terms (“sodium-glucose co-transporter 2 inhibitors” OR “SGLT2i” OR “canagliflozin” OR “JNJ 28431754” OR “dapagliflozin” OR “BMS 512148” OR “empagliflozin” OR “BI 10773” OR “ertugliflozin” OR “PF04971729” OR “sotagliflozin” OR “LX4211”) and (“chronic kidney disease” OR “CKD” OR “Renal Insufficiency”) were used. References from review articles, recent meta-analyses, and selected clinical trials were also manually searched.

### Inclusion and exclusion criteria

Eligibility criteria required: (1) randomized double-blind placebo-controlled trials; (2) trials comparing SGLT2i with placebo in CKD patients (eGFR < 90 mL/min/1.73 m^2^) with or without T2DM; (3) cardiac arrest, VT, VFL, VF, and second-degree or third-degree AVB were clearly reported in the SGLT2i and placebo groups. Excluded criteria included: (1) lack of data on any of the above-mentioned arrhythmias; (2) the research populations were not CKD patients; (3) non-placebo control.

### Study selection and outcome of interest

First, the titles and abstracts of articles were screened by two authors (Q.Y. and Y.J.) independently to determine whether they were related to our meta-analysis. Second, Full texts of possible eligible studies were reviewed by two authors (Q.Y. and Y.J.). Third, if there were disagreements, the third author (Z.L.) would make the final decision after discussing with the previous two authors.

The primary outcome was the incidence of cardiac arrest, VA, and AVB in the SGLT2i and placebo groups. Subgroup analyses were performed in different SGLT2i, different types of VA, and different degrees of AVB.

### Data extraction and quality assessment

The following data from each trial were extracted: 1) the name of the randomized controlled trial (RCT) or first author, publication year, and registration number; 2) the number of populations in each group; 3) follow-up duration, interventions, and demographic data; 4) key inclusion and exclusion criteria related. All data were obtained from Clinicaltrials.gov. The Cochrane tool for assessing risk of bias was used for the quality assessment of the trials.

### Statistical analysis

Risk ratios (RRs) and 95% confidence intervals (CIs) were calculated by a random-effects model, due to the limited included trials. Statistical heterogeneity was evaluated using the *X^2^*-test and *I^2^*. The *X^2^*-test *P* value greater than 0.10 and *I^2^* less than 50% represents low heterogeneity; otherwise, it indicates high heterogeneity. The *P*-value threshold for statistical significance was set at 0.05 for the effect sizes. The statistical analyses were performed using Review Manager 5.3. In addition, publication bias was evaluated by Begg's and Egger's tests using Stata software 15.0.

## Results

### Characteristics of the included studies

**Figure 1:**
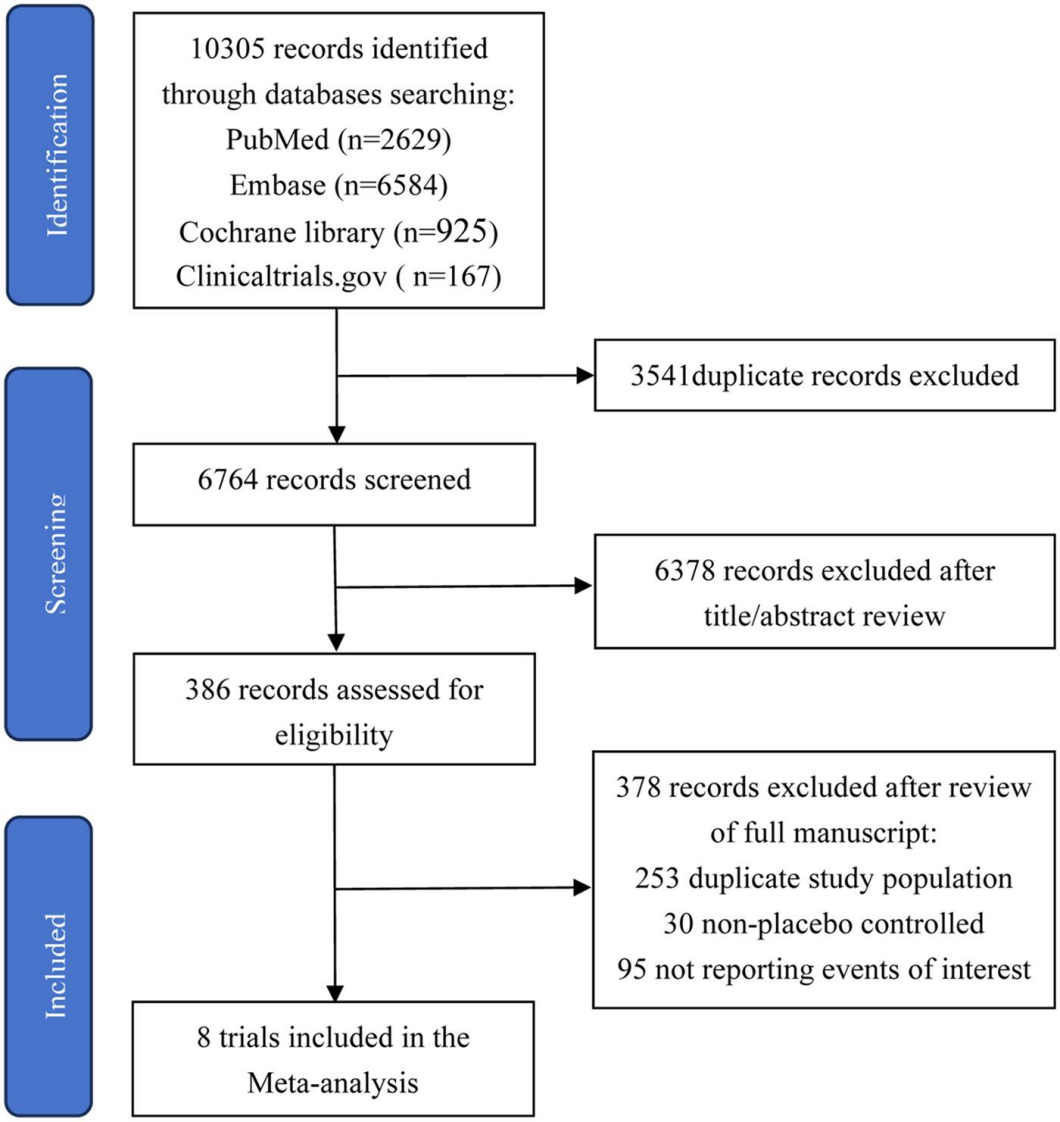
Summary of the study selection and exclusion.

8 RCTs (10–17) were identified for inclusion in the analysis. The selective process of articles is summarized in Figure 1. The baseline characteristics of trials are shown in Table 1, and the key inclusion and exclusion criteria are shown in Tables 2, 3.

**Table 1:** Baseline characteristics of all trials included in the meta-analysis.

Study, year	NCT number	Number of patients SGLT2i/placebo	Mean age (year)	Male (%)	Population	eGFR (mL/min/1.73 m^2^)	Follow-up duration	Interventions	References
EMPA-REG RENAL, 2014	NCT01164501	738 419/319	63.9	58.3	T2DM and CKD	NA	65 weeks	Empagliflozin 10 and 25 mg	(10)
Yale et al., (2014)	NCT01064414	269 179/90	68.5	60.6	T2DM and CKD	39.4 ± 6.9	52 weeks	Canagliflozin 100 and 300 mg	(11)
CREDENCE, 2019	NCT02065791	4401 2,200/2,197	63	66.1	T2DM and CKD	56.2 ± 18.2	4.6 years	Canagliflozin 100 mg	(12)
DAPA-CKD, 2020	NCT03036150	4,298 2,149/2,149	61.8	66.9	CKD	43.1 ± 12.3	39.2 months	Dapagliflozin 10 mg	(13)
SCORED, 2021	NCT03315143	10,577 5,291/5,286	68.3	55.1	T2DM and CKD	44.3 ± 10.7	30 months	Sotagliflozin 200 mg	(14)
SOTA-CKD4, 2021	NCT03242018	277 184/93	67.4	48.7	T2DM and CKD	24.0 ± 4.0	60 weeks	Sotagliflozin 200 and 400 mg	(15)
EMPA-KIDNEY, 2023	NCT03594110	6,609 3,304/3,305	63.3	66.8	CKD	37.3 ± 14.5	39 months	Empagliflozin 10 mg	(17)
SOTA-CKD3, 2023	NCT03242252	787 527/260	69.5	56.4	T2DM and CKD	45.0 ± 8.1	60 weeks	Sotagliflozin 200 and 400 mg	(16)

SGLT2i, sodium-glucose co-transporter-2 inhibitors; T2DM, type 2 diabetes mellitus; CKD, chronic kidney disease; eGFR, estimated glomerular filtration rate; NA, not available.

**Table 2:** Key inclusion criteria related to renal function and blood glucose of RCT in the meta-analysis.

Study, year	NCT number	Inclusion criteria for renal function	Inclusion criteria for blood glucose
EMPA-REG RENAL, 2014	NCT01164501	eGFR ≥ 15 to < 90 mL/min/1.73 m^2^	T2DM (HbA1c 7.0%–10.0%)
Yale et al., 2014 (11)	NCT01064414	eGFR ≥ 30 to < 50 mL/min/1.73 m^2^	T2DM (HbA1c 7.0%–10.5%)
CREDENCE, 2019	NCT02065791	eGFR ≥ 30 to < 90 mL/min/1.73 m^2^ UACR > 300 to ≤ 5000 mg/g	T2DM (HbA1c 6.5%–12.0%)
DAPA-CKD, 2020	NCT03036150	eGFR ≥ 25 to ≤ 75 mL/min/1.73 m^2^ UACR ≥ 200 to ≤ 5,000 mg/g	With or without T2DM
SCORED, 2021	NCT03315143	eGFR ≥ 25 to ≤ 60 mL/min/1.73 m^2^	T2DM (HbA1c ≥ 7.0%)
SOTA-CKD4, 2021	NCT03242018	eGFR ≥ 15 to ≤ 30 mL/min/1.73 m^2^	T2DM (HbA1c 7.0%–11.0%)
EMPA-KIDNEY, 2023	NCT03594110	1) eGFR ≥ 20 to < 45 mL/min/1.73 m^2^ 2) eGFR ≥ 45 to < 90 mL/min/1.73 m^2^ with UACR ≥200 mg/g	Without diabetes
SOTA-CKD3, 2023	NCT03242252	eGFR ≥ 30 to ≤ 60 mL/min/1.73 m^2^	T2DM (HbA1c 7.0%–11.0%)

eGFR, estimated glomerular filtration rate; UACR, urinary albumin creatinine ratio; T2DM, type 2 diabetes mellitus; HbA1c, glycated hemoglobin.

**Table 3:** Key exclusion criteria related to predisposition towards CKD and arrhythmias of RCT in the meta-analysis.

Study, year	NCT number	Exclusion criteria
EMPA-REG RENAL, 2014	NCT01164501	Uncontrolled hyperglycaemia (glucose level >13.3 mmol/L after an overnight fast); renal transplant; eGFR <15 mL/min/1.73m^2^; requirement for chronic or acute dialysis; history of ACS, stroke, or TIA within 3 months of screening; liver disease; cancer within the past 5 years; gastrointestinal surgery in the past 2 years; and treatment with antiobesity drugs within 3 months of screening or any intervention leading to unstable bodyweight at screening.
Yale et al., 2014 (11)	NCT01064414	Repeated fasting plasma glucose >15.0 mmol/L during the pretreatment phase; T1DM; renal disease that required immunosuppressive therapy, dialysis, or transplant; nephrotic syndrome or inflammatory renal disease; or ACS, revascularization procedure, or cerebrovascular accident within 3 months before screening.
CREDENCE, 2019	NCT02065791	History of DKA or T1DM, had been treated with immunosuppression for kidney disease, or had a history of dialysis or kidney transplantation; known significant liver disease; current or history of NYHA Class IV HF; blood potassium level >5.5 mmol/L during Screening.
DAPA-CKD, 2020	NCT03036150	T1DM, polycystic kidney disease, lupus nephritis, or ANCA-associated vasculitis; receiving immunotherapy for primary or secondary kidney disease within 6 months before enrollment; history of organ transplantation; NYHA class IV HF at the time of enrollment; ACS, stroke, or TIA within 12 weeks before enrollment.
SCORED, 2021	NCT03315143	Antihyperglycemic treatment has not been stable within 12 weeks before screening; planned coronary procedure or surgery after randomization.
SOTA-CKD4, 2021	NCT03242018	T1DM; renal disease requiring treatment with immunosuppressive therapy or dialysis within the past 12 months or the expectation that dialysis would be needed during the study; uncontrolled high blood pressure, severe anemia, severe cardiovascular problems, active cancer, or other conditions that may result in a short life expectancy.
EMPA-KIDNEY, 2023	NCT03594110	Maintenance dialysis, functioning kidney transplant, or scheduled living donor transplant; polycystic kidney disease; previous or scheduled bariatric surgery; symptomatic hypotension; ALT or AST >3xULN at Screening; any intravenous immunosuppression therapy in the last 3 months; or anyone currently on >45 mg prednisolone (or equivalent).
SOTA-CKD3, 2023	NCT03242252	T1DM; uncontrolled high blood pressure; history of DKA within 12 weeks before screening or reversible renal failure or severe hypoglycemia within 6 months of screening.

eGFR, estimated glomerular filtration rate; ACS, acute coronary syndrome; TIA, transient ischemic attack; T1DM, type 1 diabetes mellitus; DKA, diabetic ketoacidosis; NYHA, New York heart association; HF, heart failure.

**Figure 2:**
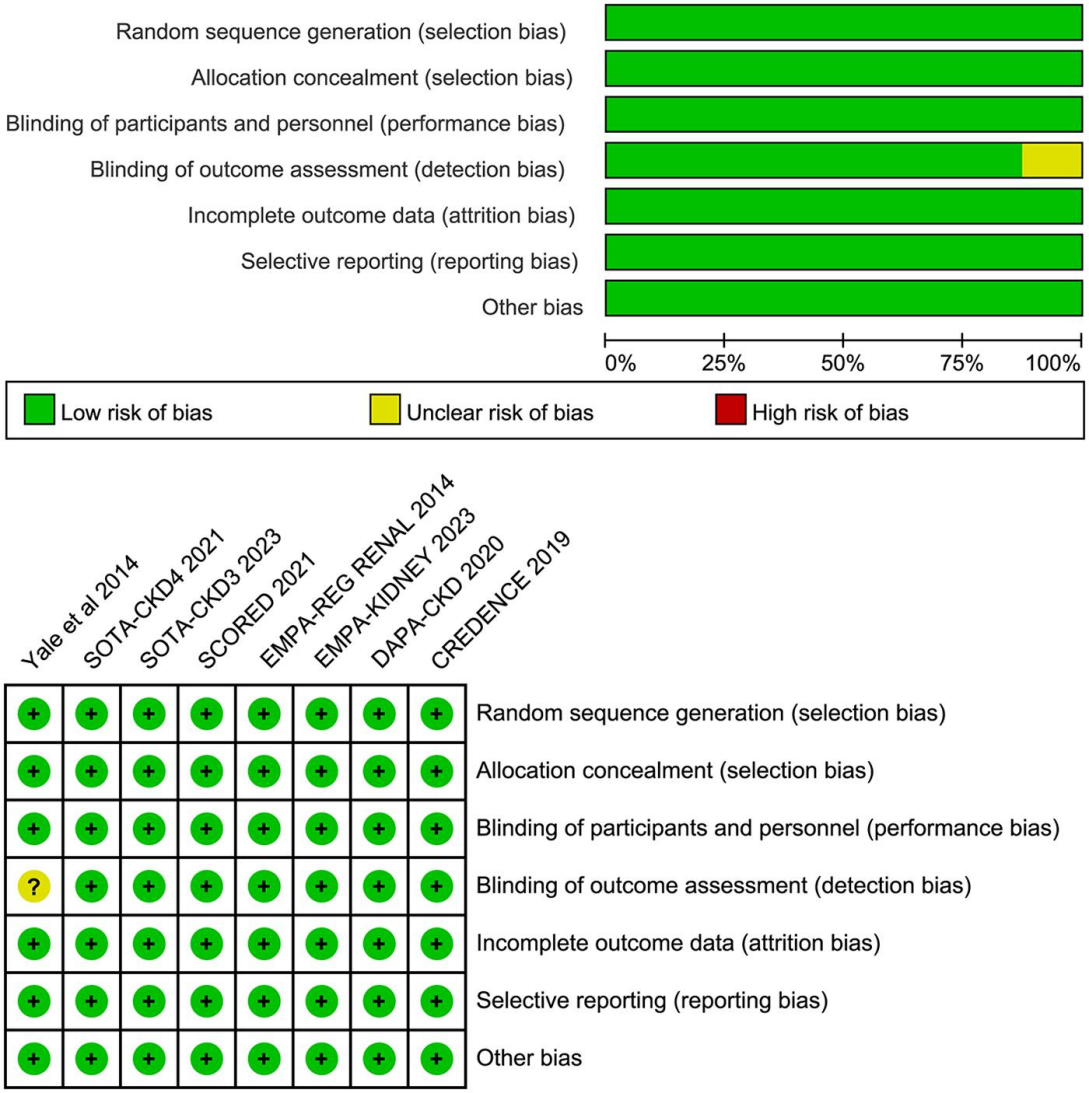
Effect of SGLT2i on cardiac arrest in patients with CKD.

The risk of bias graph and risk of bias summary figure were used to present the quality assessment of the included RCTs in Figure 2. All included RCTs have high methodological quality.

### Cardiac arrest

**Figure 3:**
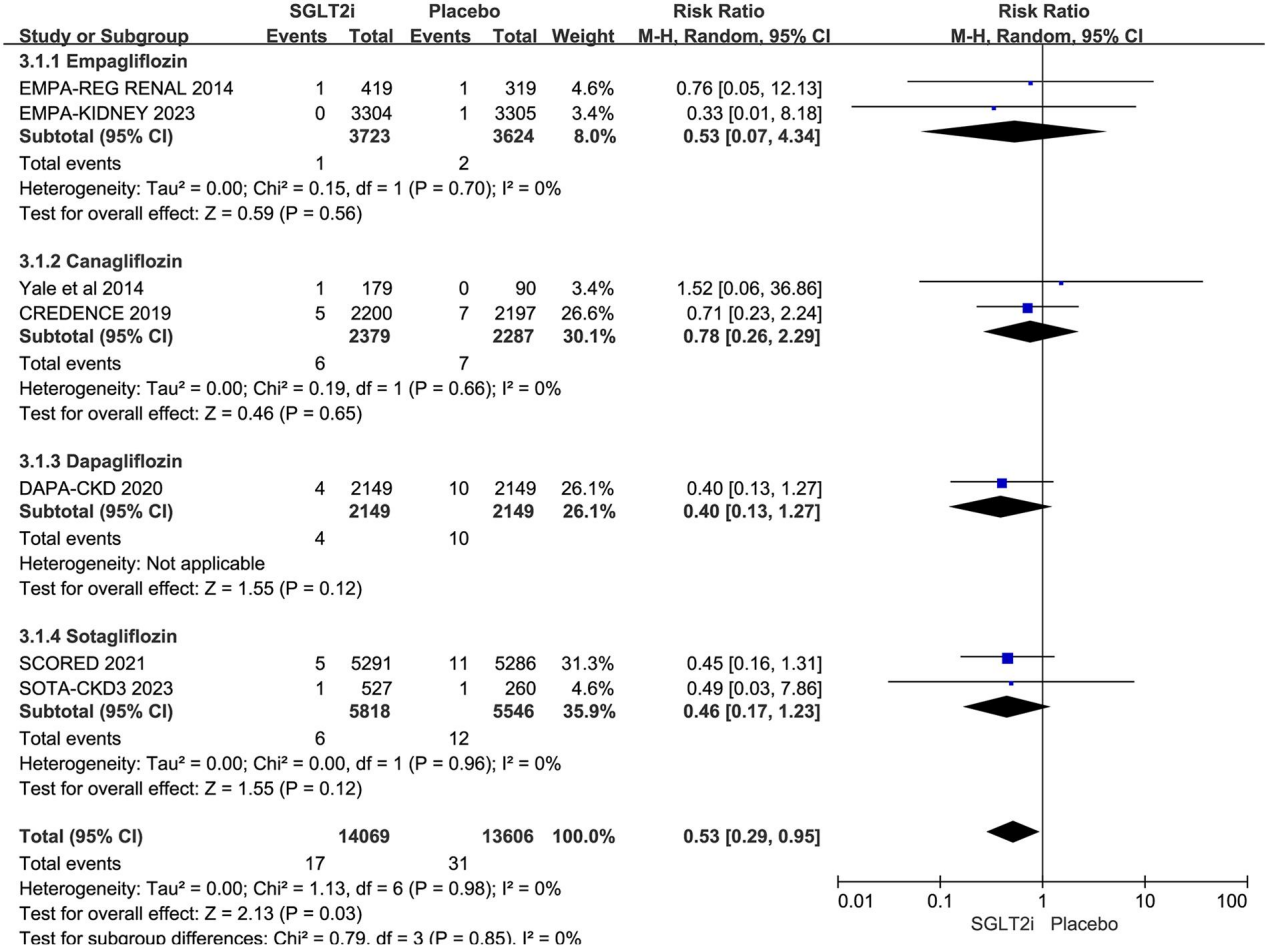
Subgroup analysis of cardiac arrest based on different types of SGLT2i.

The trial of SOTA-CKD4 (15) didn't report cardiac arrest in the SGLT2i and placebo groups. Therefore, 7 of 8 RCTs reported cardiac arrest. It occurred in 17 of 14,069 patients in the SGLT2i group and 31 of 13,606 patients in the placebo group. The result showed that SGLT2i reduced the risk of cardiac arrest in CKD patients (0.12% vs. 0.23%; RR 0.53, 95% CI 0.29–0.95, *P* = 0.03), with no substantial heterogeneity (*P* = 0.98, *I*^2^ = 0%). The results are shown in Figure 3.

Of the 7 trials, 2 with empagliflozin (10, 17), 2 with canagliflozin (11, 12), 1 with dapagliflozin (13), and 2 with sotagliflozin (14, 16). Subgroup analysis was performed based on different SGLT2i, and the result did not indicate which SGLT2i had a preventive effect on cardiac arrest (empagliflozin: RR 0.53, 95% CI 0.07–4.34, *P* = 0.56; canagliflozin: RR 0.78, 95% CI 0.26–2.29, *P* = 0.65; dapagliflozin: RR 0.40, 95% CI 0.13–1.27, *P* = 0.12; sotagliflozin: RR 0.46, 95% CI 0.17–1.23, *P* = 0.12). The results are also shown in Figure 3.

Of the 7 trials, the eGFR ranged from 15 to 90 mL/min/1.73 m^2^. For the reason that all the 7 trials didn't group patients based on eGFR (<30 vs. ≥30 mL/min/1.73 m^2^) or stage of CKD, we were unable to conduct subgroup analysis based on these.

### Ventricular arrhythmia

**Figure 4:**
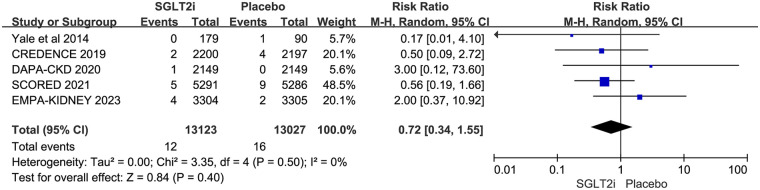
Effect of SGLT2i on VA in patients with CKD.

5 RCTs reported VA, including VT, VFL, and VF. VA occurred in 12 of 13,123 patients in the SGLT2i group and 16 of 13,027 patients in the placebo group. The result showed that SGLT2i did not affect VA in CKD patients (0.09% vs. 0.12%; RR 0.72, 95% CI 0.34–1.55, *P* = 0.40), with no substantial heterogeneity (*P* = 0.50, *I*^2^ = 0%). The results are shown in Figure 4.

**Figure 5:**
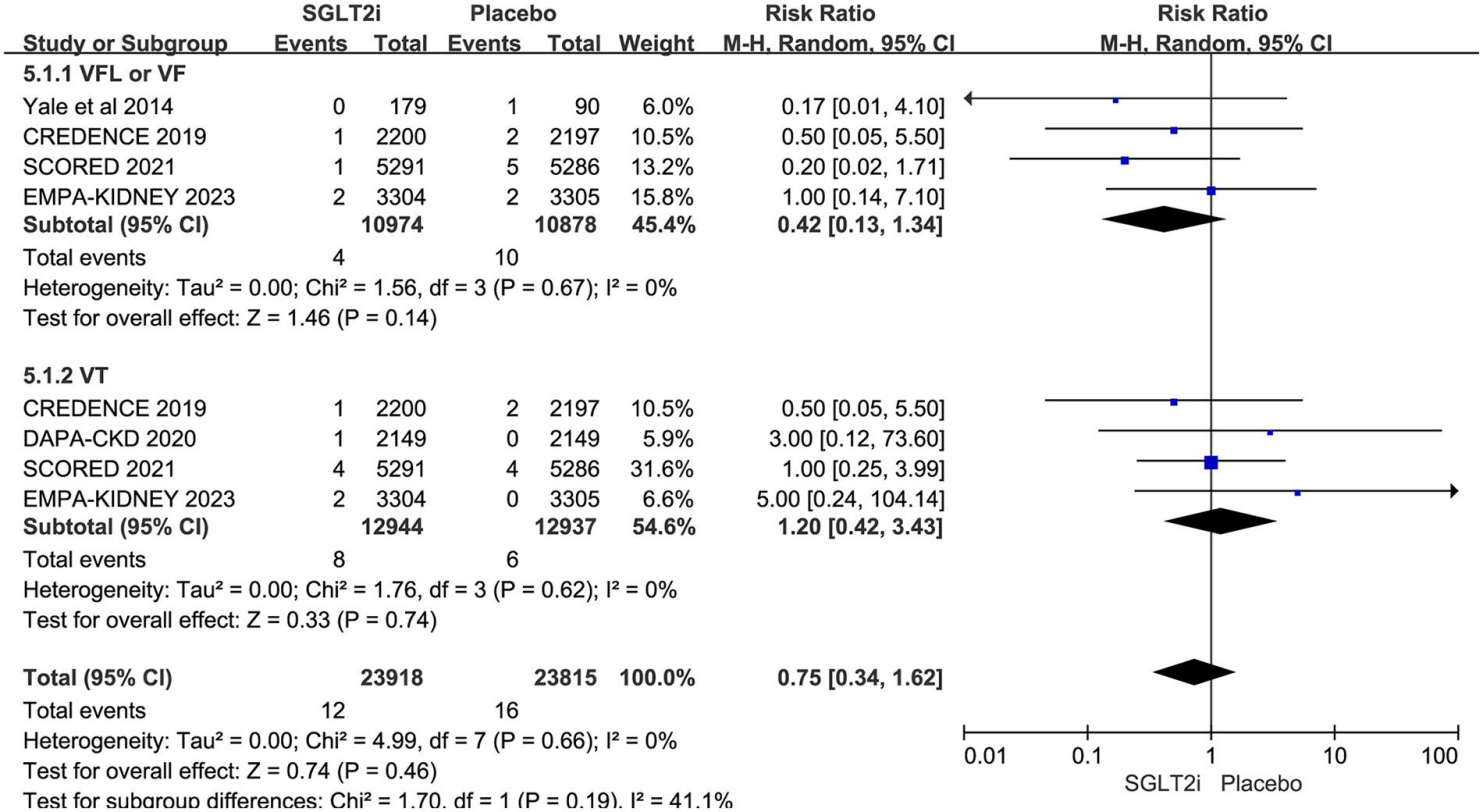
Subgroup analysis based on different types of SGLT2i.

Of the 5 trials, 4 reported VT (12–14, 17), 1 reported VFL (14), and 4 reported VF (11, 12, 14, 17). As VFL is only reported in one trial, it was analyzed with VF. VFL/VF occurred in 4 of 10,974 patients in the SGLT2i group and 10 of 10,878 patients in the placebo group. VT occurred in 8 of 12,944 patients in the SGLT2i group and 6 of 12,937 patients in the placebo group. Subgroup analysis based on different types of VA showed that SGLT2i did not reduce the risks of either VFL/VF (0.036% vs. 0.092%; RR 0.42, 95% CI 0.13–1.34, *P* = 0.14) or VT (0.062% vs. 0.046%; RR 1.20, 95% CI 0.42–3.43, *P* = 0.74) in CKD patients. However, the risk of VFL/VF was borderline significant. The results are shown in Figure 5.

### Atrioventricular block

**Figure 6:**
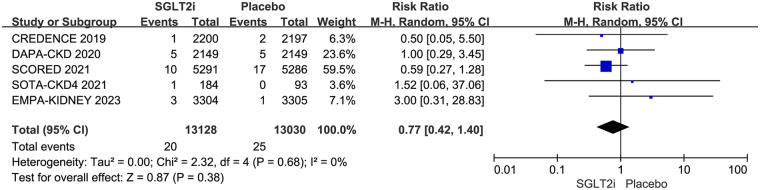
Effect of SGLT2i on AVB in patients with CKD.

5 RCTs reported AVB, including second-degree and third-degree AVB. AVB occurred in 20 of 13,128 patients in the SGLT2i group and 25 of 13,030 patients in the placebo group. The result showed that SGLT2i did not affect AVB in CKD patients (0.15% vs. 0.19%; RR 0.77, 95% CI 0.42–1.40, *P* = 0.38), with no substantial heterogeneity (*P* = 0.68, *I*^2^ = 0%). The results are shown in Figure 6.

**Figure 7:**
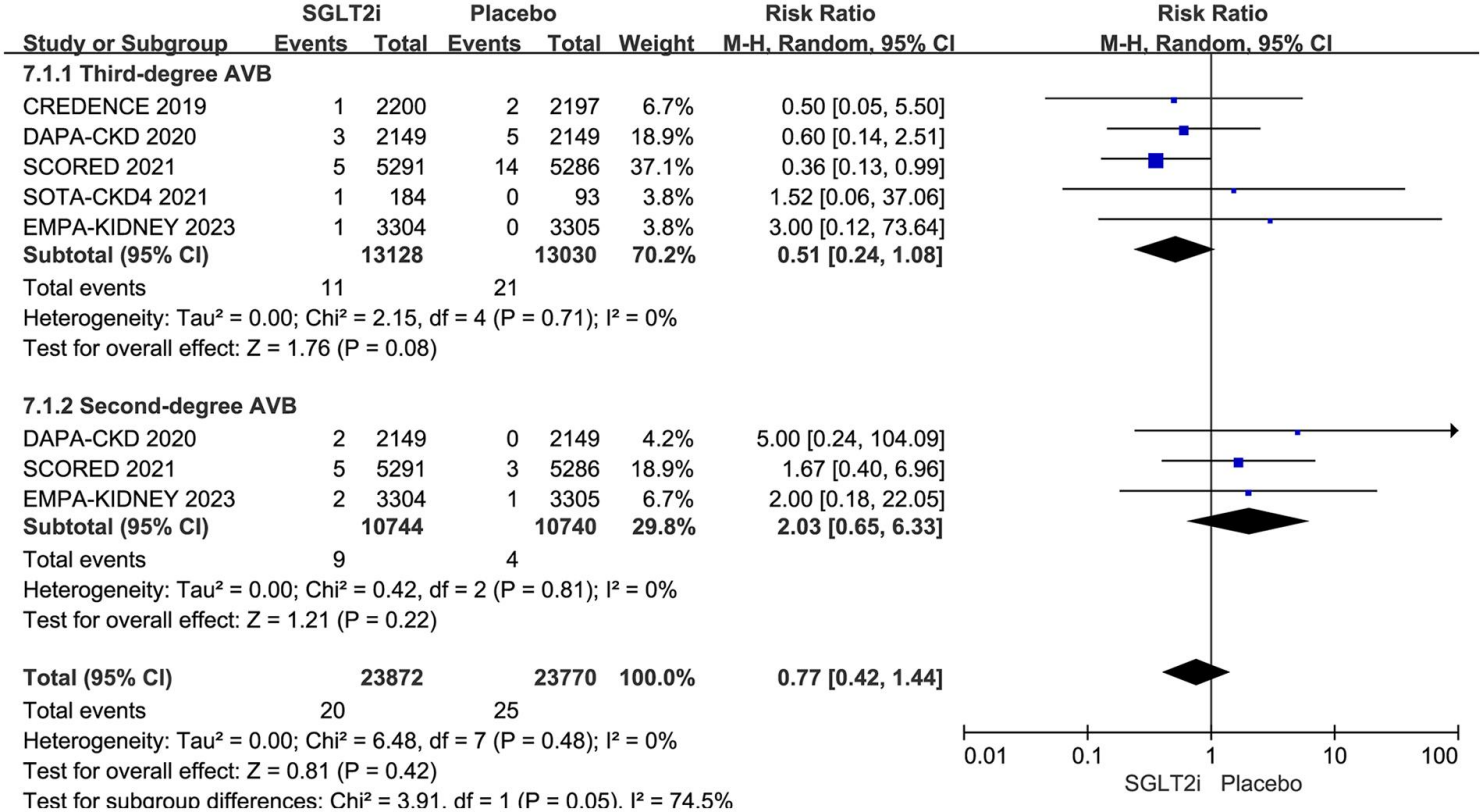
Subgroup analysis based on different degrees of AVB.

Of the 5 trials, 5 reported third-degree AVB (12–15, 17), and 3 reported second-degree AVB (13, 14, 17). Third-degree AVB occurred in 11 of 13,128 patients in the SGLT2i group and 21 of 13,030 patients in the placebo group. Second-degree AVB occurred in 9 of 10,744 patients in the SGLT2i group and 4 of 10,740 patients in the placebo group. Subgroup analysis based on different degrees of AVB showed that SGLT2i did not reduce the risks of either third-degree (0.084% vs. 0.161%; RR 0.51, 95% CI 0.24–1.08, *P* = 0.08) or second-degree (0.084% vs. 0.037%; RR 2.03, 95% CI 0.65–6.33, *P* = 0.22) in CKD patients. However, the risk of third-degree AVB was borderline significant. The results are shown in Figure 7.

### Publication bias

Begg's and Egger's tests were used to evaluate publication bias. No significant risk of publication bias was found in cardiac arrest (Begg, *P* = 0.548; Egger, *P* = 0.490), VA (Begg, *P* = 0.806; Egger, *P* = 0.794), and AVB (Begg, *P* = 0.806; Egger, *P* = 0.263).

## Discussion

CKD is a worldwide public health problem, with the prevalence of 13.4% globally (1). The leading cause of death for CKD is cardiac arrest and SCD. Patients, no matter with earlier stage CKD or chronic kidney failure, all had a significantly increased risk of SCD compared with the general population (18, 19), and more than two-thirds of mortality in advanced CKD (stage 4 and 5) is caused by SCD (1).

The majority of SCD is caused by life-threatening arrhythmias, such as ventricular arrhythmia (VA) and severe bradyarrhythmia (1, 2). VA includes VT, VFL, and VF. Bradyarrhythmia includes severe sinus bradycardia and AVB, which are prone to secondary VA and eventually lead to cardiac arrest and SCD.

SGLT2i, as a new glucose-lowering drug, has been proven to have protective effects on the heart and kidney (3). Initially, considering the possible electrolyte imbalance caused by osmotic diuresis, SGLT2i was thought to potentially favor cardiac arrhythmias. Surprisingly, clinical research (20) showed that SGLT2i not only did not increase the risk of arrhythmias but also reduced the prevalence of atrial fibrillation and cardiac arrest in T2DM patients, while its effect on VA was not conclusive. In recent years, some RCTs evaluating the effect of SGLT2i on CKD patients were carried out, and many types of arrhythmias as adverse events were reported, which enables us to perform a meta-analysis to investigate whether SGLT2i could reduce the risk of arrhythmias in CKD patients. It should be noted that patients included in the trials rarely only had CKD without T2DM, and most of them had both CKD and T2DM, as shown in Table 2.

Recently, Hageen et al.'s meta-analysis (21) showed that SGLT2i combined with aldosterone inhibitors could reduce albuminuria and slow CKD progression. Our previous meta-analysis showed that SGLT2i could reduce the risk of atrial fibrillation and flutter in CKD patients (6). However, whether SGLT2i could reduce the risk of life-threatening arrhythmias such as cardiac arrest, VA, and AVB in CKD patients was not clear. In recent years, some meta-analyses evaluated the effect of SGLT2i on VA and cardiac arrest in the overall population (7, 8, 22), type 2 diabetes mellitus (T2DM) patients (23), and heart failure (HF) patients (24), but none of them specifically focused on CKD patients. Li et al.'s meta-analysis (7) included 39 trials, and only 8 of them focused on CKD patients. They evaluated the effect of SGLT2i on the risk of VT, VF, and sinus bradycardia, but did not evaluate the effect on cardiac arrest and AVB, and did not perform subgroup analyses based on different populations. Therefore, we performed this meta-analysis, which included 8 RCTs of CKD patients, and hoped to provide more options for the treatment of life-threatening arrhythmias in CKD patients.

None of the included RCTs reported SCD events. Cardiac arrest was reported in 7 RCTs, including 27,675 patients with CKD who received SGLT2i (empagliflozin, canagliflozin, dapagliflozin, and sotagliflozin) or placebo. The result showed that SGLT2i significantly reduced the risk of cardiac arrest in patients with CKD by 47.83% compared with placebo (0.12% vs. 0.23%). Previous meta-analyses did not find this preventive effect of SGLT2i on cardiac arrest in the overall population (OR 0.94, 95% CI 0.72–1.23, *P* = 0.67) (22) and T2DM patients (RR 0.88, 95％CI 0.66–1.18, *P* = 0.39) (23). Wang et al.'s meta-analysis (8) also showed SGLT2i could reduce the risk of cardiac arrest in CKD patients, but only 3 RCTs were included. Subgroup analysis based on different SGLT2i tried to discover which gliflozins had a preventive effect, but the result showed that none of the four gliflozins reduced the risk of cardiac arrest. It might be related to the fact that there are only 1–2 RCTs in each type of gliflozin.

Our meta-analysis included 5 RCTs and evaluated VA in 26,150 patients with CKD. VA includes VT, VFL, and VF. As VFL was only reported in one trial, it was analyzed with VF. The result showed that SGLT2i did not reduce the risks of VA, VF/VFL, and VT, but there was a potentially effective trend of SGLT2i on preventing VFL/VF (RR 0.42, 95% CI 0.13–1.34, *P* = 0.14). The SGLT2i group had a 60.87% lower risk of VFL/VF compared to the placebo group, but did not reach statistical superiority (0.036% vs. 0.092%). Previous meta-analyses showed that SGLT2i also could not reduce the risk of VA in T2DM patients (RR 0.94, 95％CI 0.71–1.26, *P* = 0.69) (23), but could reduce the risk of VA in HF patients (RR 0.85, 95％CI 0.74–0.98, *P* = 0.02) (25) which included 22 RCTs.

Some previous meta-analyses evaluated the effect of SGLT2i on bradyarrhythmia and sinus bradycardia, and rarely focused on AVB. Actually, second-degree and third-degree AVB are more serious than other bradyarrhythmias. If they are not caused by reversible factors, the vast majority of them require permanent pacemaker implantation. First-degree AVB was not included for the reason that most of them were not severe, and they were rarely reported in RCTs. Our meta-analysis included 5 RCTs and evaluated second-degree and third-degree AVB in 26,158 patients with CKD. The result showed that SGLT2i did not reduce the risk of AVB, neither second-degree nor third-degree AVB, but there was a potentially effective trend of SGLT2i on preventing third-degree AVB (RR 0.51, 95% CI 0.24–1.08, *P* = 0.08). The SGLT2i group had a 48.83% lower risk of third-degree AVB compared to the placebo group, but did not reach statistical superiority (0.084% vs. 0.161%). Sinus bradycardia was only reported in 3 RCTs, and the incidence rate is extremely low in each group. Therefore, it was not analyzed in our article.

It is essential to emphasize that cardiac arrest, VA, and AVB were reported as adverse events rather than prespecified endpoints in all included RCTs. This may lead to under-reporting and misclassification bias. Previous study (26) conducted on French patients with stage 3–5 CKD found that the incidence of SCD was 0.37% during a median follow-up period of 5 years, which was significantly higher than the incidence of cardiac arrest in our analysis (SGLT2i group: 0.12%; placebo group: 0.23%). Patients with cardiac arrest caused by VA or AVB may be wrongly attributed to cardiac arrest caused by other diseases or unknown reasons. Because the diagnosis of VA and AVB requires an electrocardiogram or electrocardiographic monitoring. When they were reported as adverse events, it was difficult to obtain these data.

Our meta-analysis showed that SGLT2i reduced the risk of cardiac arrest in CKD patients, but had no effect on specific types of arrhythmias. However, the risks of VF and third-degree AVB between the SGLT2i and placebo groups were borderline significant, which meant that SGLT2i might be effective for VF and third-degree AVB. There are two reasons for the negative results. First, as mentioned above, arrhythmic events were all reported as adverse events, which meant that some events had been underreported. VF and third-degree AVB were more likely to be underreported than cardiac arrest. Second, the incidences of life-threatening arrhythmias, such as VF and third-degree AVB, are significantly low. In our subgroup analyses, only 4 or 5 RCTs reported VF and third-degree AVB, and the number of RCTs is very limited. So, more large-scale and long-term follow-up trials are needed to evaluate the effects of SGLT2i on specific types of arrhythmias.

Currently, SGLT2i is widely used in CKD patients with or without T2DM, as it has been proven to have significant protective effects on the kidneys and heart. However, these proven protective effects don't include the fact that SGLT2i could reduce the risk of cardiac arrest in CKD patients. We all know that once cardiac arrest occurs, it is usually fatal. Therefore, our meta-analysis is of great significance, and it expands the potential protective effects of SGLT2i on CKD patients. Of course, more clinical trials are needed to confirm the effect of SGLT2i, especially for patients with different stages of CKD.

CKD increases the risk of arrhythmia due to electrolyte imbalances, inflammation, oxidative stress, uremic cardiomyopathy, and autonomic neuropathy (27). The effect of SGLT2i on cardiac arrest has been less well studied. The potential mechanisms of SGLT2i on cardiac arrest may include the following aspects (4, 5, 28). First, increased sympathetic activity plays an important role in the development and maintenance of cardiac arrhythmias (28, 29). SGLT2i could directly act on the central nervous system and inhibit the discharge activity of neurons in the ventrolateral region of the head end of the medulla oblongata. This area is the key center for sympathetic nerve output, and the decrease in its excitability can directly reduce the sympathetic nerve outflow throughout the body (30). In addition, SGLT2i improved the internal environment of the kidney by alleviating hypoxia, oxidative stress, and inflammation, and restored the abnormal renal afferent nerve signals, thereby reducing the sympathetic output in the central nervous system (30, 31). Second, the circulatory pressure overload and uremic environment resulting from CKD stimulate cardiac remodeling (4). Myocardial fibrosis and hypertrophy are characteristic changes in CKD patients (1), which increase cardiac automaticity, trigger focal activity and the formation of reentry circuits associated with the development and progression of cardiac arrhythmias. SGLT2i could reduce blood volume, ameliorate myocardial inflammation and oxidative stress, thereby preventing cardiac remodeling (4, 28). Third, contraction of cardiac muscle requires a large amount of energy. Arrhythmias are closely related to changes in metabolic activity (32). SGLT2i promote gluconeogenesis, fatty acid oxidation, and ketone body synthesis (5), which not only supplies energy but also resists oxidative and inflammatory injury (33), thereby producing benefits on cardiac energetics and adverse cardiac remodeling.

### Limitations

There are several limitations in our meta-analysis. First, all arrhythmic events were reported as adverse events rather than primary or secondary outcomes, which may lead to under-reporting bias. Second, patient-level data were not available, and not all trials reported baseline comorbidity and background therapy, especially the use of anti-arrhythmic drugs. Third, the incidences of arrhythmia events are relatively low, and more large-scale and long-term follow-up trials are needed to evaluate the anti-arrhythmic effect of SGLT2i.

## Conclusion

In our analysis, SGLT2i could reduce the risk of cardiac arrest in CKD patients, but had no effect on specific types of life-threatening arrhythmias, such as VT, VF/VFL, second-degree and third-degree AVB.

## Data Availability

The original contributions presented in the study are included in the article/Supplementary Material, further inquiries can be directed to the corresponding author.
